# Small impact of the host plant physiological state on the genome formula of a nanovirus

**DOI:** 10.1371/journal.ppat.1014641

**Published:** 2026-09-21

**Authors:** Mélia Bonnamy, Andy Brousse, Prune Lacôte Popovic, Yannis Michalakis, Stéphane Blanc

**Affiliations:** 1 PHIM, Univ Montpellier, IRD, CIRAD, INRAE, Institut Agro, Montpellier, France; 2 MIVEGEC, CNRS, IRD, Univ Montpellier, Montpellier, France; University of Tübingen: Eberhard Karls Universitat Tubingen, GERMANY

## Abstract

The genome formula refers to the specific and reproducible pattern of relative accumulation of genome segments in within-host populations of viruses with segmented genomes. It has been proposed that the genome formula may have an adaptive function by allowing rapid adaptation of viral genes’ expression, through variation in their relative copy number, in response to a changing environment. Consistently, the faba bean necrotic stunt virus (FBNSV) genome formula has been demonstrated to be strongly dependent on the host genotype, since it changes significantly when the same viral isolate moves from one host species to another. However, the cellular environment of the virus can also be modulated by the growing conditions and/or stresses that impact on plant physiology. In this study, we thus cultivated *Vicia faba* plants under various biotic and abiotic stressing conditions with the aim of modifying the plant physiology in various and complementary ways and assess its potential impact on the genome formula. Although the plants exhibited phenotypes altered by the tested growing conditions, the total FBNSV accumulation was not impacted by the physiological changes in the plant except by aphid infestation which reduced it. Importantly, in contrast to the drastic change observed when switching hosts, the genome formula showed little or no variation across growing conditions for all the tested stressors. Our results thus indicate that the genome formula is marginally influenced by the physiological state of the host.

## Author summary

Viruses depend on their host cell to complete their life cycle. However, the cellular environment can fluctuate depending on the host’s physiological state, thereby constraining the virus to adapt the expression of its genes to this new cellular context. Viruses with segmented genomes (monopartite segmented and multipartite viruses) distribute their genetic information across different molecules called genome segments. Within the host, the different genome segments can accumulate at distinct relative frequencies, resulting in a variable number of copies of the genes carried by the respective segments. Therefore, it has been proposed that the fragmentation of the genome across different segments could enable rapid adaptation of viral gene expression to the cellular environment by modulating their copy number. However, under the physiological variations tested here, our study shows that changes in the physiological state of the host plant caused by various stressors have little to no impact on the relative accumulation of the different genome segments of the multipartite FBNSV. This observation contrasts with the extensive changes previously reported following host switch.

## Introduction

Organisms must adapt their gene expression to their environment. This adaptation can be achieved by genome modifications, such as mutations in coding or regulatory DNA sequences, or by transcriptional and post-transcriptional regulation mechanisms, such as DNA methylation or protein ubiquitination respectively. In the case of genome modifications, adaptive mutation rate can be low, selection to fixation can be slow, and thus these processes may sometimes fail to allow rapid adaptation of gene expression [1,2]. Another phenomenon, considered to be a fast and coarse response, is the regulation of gene expression by copy number variation and the associated gene-dosing effect (CNV) [3,4]. The CNV mechanism is reversible and allows rapid adaptation of gene expression to changing environments [5]. It relies on gene amplification and contraction and has been named genomic or genetic accordion [4,6]. Adaptive CNV is a ubiquitous mechanism, empirically validated in various systems such as insects [7,8], fungi [9], bacteria [4], and large double-stranded DNA monopartite viruses that can easily accommodate large genome size variations [6].

In viruses with a segmented genome, the different genome segments reproducibly accumulate at different relative frequencies in the host (reviewed in [10]). This phenomenon, known as genome formula [11], is believed to play a role analogous to CNV or genomic accordion [11–15]. It is well established that the genome formula is affected by the host genotype, since the relative frequency of genome segments changes upon host switching [11,12,16–19]. The genome formula can also be influenced by other environmental components, such as the infected organ or the presence of satellites [10]. However, the importance of the physiological state of a given host genotype – which is dependent on the host environment – in modulating the genome formula has not been studied. Viruses rely on their host cell machineries to complete their infection cycle and changes in the host’s physiology, particularly those associated with biotic or abiotic stresses, could alter the interactions between the virus and cellular components. Such modifications could be accommodated through changes of the genome formula, in a manner analogous to CNV, as observed upon host switching.

To determine whether physiological changes in the host plant impact the genome formula, we used the faba bean necrotic stunt virus (FBNSV; family *Nanoviridae*, genus *Nanovirus*), an aphid transmitted virus infecting vascular tissues of many legume plants. FBNSV is a multipartite virus whose genome is divided into eight single-stranded DNA (ssDNA) segments (C, M, N, R, S, U1, U2, U4). Each genome segment is encapsidated individually and encodes a unique protein [20,21]. We estimated the relative frequency of the FBNSV segments in faba beans (*Vicia faba*), the host on which this virus was originally isolated from, grown under a variety of culture conditions. We chose a relatively large panel of stressors based on their impact on the plant and on how they may interfere with the viral cycle. Indeed, while the perception of many abiotic stresses by a plant can lead to the activation of common signaling, notably calcium spiking, oxidative burst or modulation of the production of various phytohormones [22], the downstream pathways and final responses are at least partly specific [22,23]. They potentially involve different players who can differentially interfere with viral infection. Likewise, different biotic stresses may affect the viral cycle in variable ways. They can either prime or dampen plant defenses, they may mobilize resources in direct competition with the virus or, on the contrary, have a synergistic effect and facilitate infection.

Overall, our results show that, unlike host genotypes which cause major modifications [11,12], the host physiological status induced by a diversity of growing conditions and stresses appears to have only marginal effects on the FBNSV genome formula.

## Materials and methods

### Justification of the stresses tested

We tested a panel of abiotic (water deficiency, high sodium chloride (NaCl) concentration, different light intensities, different temperatures and nitrogen (N) deficiency) and biotic (co-infection with RNA or DNA viruses, aphid infestation and rhizobial symbiosis) stressors based on their impact on the plant physiology and thus the different manners they could interfere with the viral cycle. These are described below.

The signaling pathways and downstream effects on plants of abiotic stresses such as water deficit, high salt concentration and temperature are extensively documented [22,23]. Water deficiency generates hyperosmotic stress, leading to plasmolysis and detachment of the plasma membrane from the cell wall. In addition, high salt concentration results in the accumulation of ions (Na^+^ and Cl^-^) that are toxic to the cell. On the one hand, calcium ions remobilization to the cytosol caused by hyperosmosis and salt could inhibit protein trafficking and thus alter the plant’s immune system [24]. On the other hand, calcium and other divalent ions are co-factors of various enzymes and contribute to the folding of a large range of proteins, potentially impacting viral genome replication and expression, as well as virion assembly, maturation and stability [25]. Temperature can influence the physical properties of molecules, modifying RNA and ssDNA secondary structures, protein stability, conformation and function, as well as membrane properties [22,23], again potentially altering various host-virus interactions including RNA interference [26] and viral cell-to-cell movement [27]. Light intensity drastically affects photosynthesis, leading to changes of carbon source accumulation and resulting in a reallocation of resources. Low light intensity favors essential metabolic processes and reduces the production of secondary metabolites [28]. In contrast, high light intensity increases photosynthetic activity and can lead to the overproduction of reactive oxygen species (ROS). This oxidative environment activates a range of protective and detoxification mechanisms and is often associated with increased production of secondary metabolites. Finally, reactive oxygen species can also induce damage to cellular components, including nucleic acids, thereby activating the DNA break/repair machinery that is involved in the replication of nanoviruses [29].

Biotic stresses could interact with FBNSV either indirectly by modification of the plant’s physiological state and in particular its defense systems, or directly by competition for cellular resources. We here use the bean yellow mosaic virus (family *Potyviridae*, genus *Potyvirus*), a single-stranded RNA virus which attenuates the plant’s defense mechanisms upon infection, notably by suppressing post-transcriptional gene silencing [30]. Numerous cases of synergy linked to the suppression of PTGS by potyviruses have been described during co-infections with other viruses [31]. Co-infection with BYMV could therefore generate beneficial modifications for FBNSV. Conversely, the two viruses could compete for certain cellular resources, such as the plasmodesmata they both use to bidirectionally move from companion cells to sieve elements [32]. As another example, we also used the alfalfa leaf curl virus (ALCV; genus *Capulavirus*) belonging to the second family of plant-infecting ssDNA viruses, *Geminiviridae*. Member species of this family share several similarities with nanoviruses, notably regarding the proteins they encode and their replication mechanisms [20,29]. Hence, one could easily speculate on likely competition between the two viruses for cellular/nuclear factors required to achieve their infection cycle. In another range of biotic stresses, aphid infestation could indirectly interfere with FBNSV infection. Aphid punctures into plant tissues immediately activate defense mechanisms, including the reinforcement of the cell wall through callose deposition [33] and the occlusion of the phloem sieve tubes through polymerization of P proteins [34] or contraction of the forisome [35,36] blocking the sap flow. It is therefore logical to hypothesize that an aphid infestation could affect the intra- and intercellular movements of the virus. Finally, we also assessed the impact of growing plants with or without *Rhizobium* symbiosis, because this has both a biotic effect, *via* interaction with the bacteria, and an abiotic effect, *via* N nutrition. The symbiotic interaction between plants and *Rhizobium* induces a systemic response in the plant, inducing changes in defense mechanisms and altered metabolism, which have been shown to increase plant tolerance to some viral infections [37,38]. On the other hand, by converting atmospheric N into a form that can be assimilated by the plant, *Rhizobium* provides the plant with N even in nitrate deprived soils. N is an essential component of the cell, necessary for the completion of numerous metabolic and signaling functions and is thus indispensable for plant growth and development [39,40]. The impact of N nutrition on host-pathogen interactions is complex and N deficiency can have contrasting effects depending on the system: it could hinder infection by reducing the resources available to the pathogen; conversely, it could promote infection by reducing the resources allocated to the plant’s defense mechanisms [41].

### FBNSV clone and *Agrobacterium*-mediated inoculation

The FBNSV infectious clone (isolate JKI-2000) was provided by T. Timchenko, CNRS, Gif sur Yvette [42] and is composed of eight binary plasmids derived from pBIN-19, each containing a tandem repeat of one of the segments (C, M, N, R, S, U1, U2, U4). Each plasmid was transformed into one colony of *Agrobacterium tumefaciens* strain COR308.

The eight *A. tumefaciens* clones were grown separately as described earlier [11]. Bacteria from each culture were then pelleted by centrifugation and resuspended in 5 mL of MS buffer [43] containing 1 mM MES (2-morpholin-4-ylethanesulfonic acid) pH 5.5 and 200 µM acetosyringone. The resuspensions were mixed and incubated 2h at room temperature. The bacterial mixture was inoculated by infiltration on the underside of young emerging leaves.

### Plant growing conditions

#### Water deprivation.

**Fig 1:**
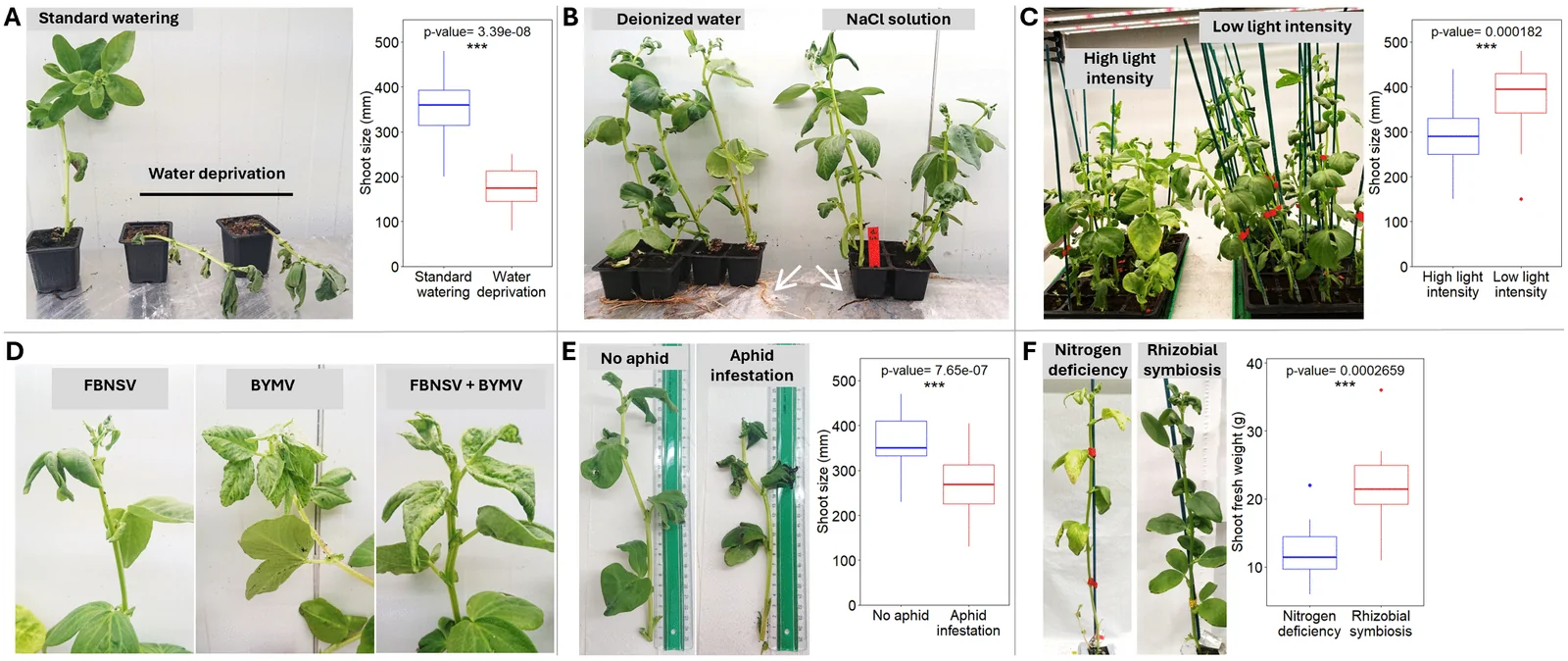
Phenotype of FBNSV-infected *V. faba* grown under different conditions. (A) water deprivation, (B) high NaCl concentration, (C) different light intensities, (D) BYMV co-infections, (E) aphid infestation and (F) nitrogen deficiency or rhizobial symbiosis. Plants were cultivated and infected as described in the Material & Methods section. Pictures and measurements were taken about 20 days (A-D and F) or 14 days (E) after FBNSV inoculation. The white arrows in B highlight decreased root development consistently observed in the plant set with salt excess versus control plants. In D, typical symptoms shown for FBNSV are inward curling of apical leaves, whereas those shown for BYMV are light and dark green mosaic. In boxplots, the central bar represents the median and the edges of the rectangle the first and third quartiles. The outer bars delineate 1.5 times the distance between the 25^th^ and 75^th^ percentile of the distribution. The dots represent the outliers. Statistically significant differences between conditions were assessed on non-transformed data based on ANOVA and Tukey HSD tests (also see Tables C and D in S1 Appendix). All statistically significant differences shown in the figure had p < 0.001 and are indicated by ***.

*V. faba* plants (var. Seville; Vilmorin) were grown in individual pots (6 cm x 6 cm x 6 cm) in a growth chamber with 13h/11h day/night photoperiod, 25°C/18°C day/night temperature. The seeds were watered abundantly after sowing to trigger germination. Nine days later, plantlets were agro-inoculated with FBNSV as described above. Plants from the water deficit modality received a second restrained watering with 10 mL of water at 5 days post-inoculation (dpi) to keep them alive until the sampling date. In parallel, plants from the control modality were watered abundantly every 3–4 days. Total DNA was extracted at 20 dpi following the protocol described below. To facilitate DNA extraction, the plants wilted under water deficit were rehydrated (and thus watered) the day preceding extraction. A batch of non-rehydrated plants was also extracted to ensure that this final watering did not significantly impact on FBNSV genome formula (Table A in S1 Appendix). Physiological changes in plants caused by water deprivation were assessed by visual observations of severe wilting and shoot size measurement at 20 dpi (Fig 1A).

#### Increased salt concentration.

*V. faba* plants were grown in a growth chamber with 13h/11h day/night photoperiod, 25°C/18°C day/night temperature. They were watered with a NaCl solution starting at 25 mM two days after sowing and increasing by 25 mM every two days up to a concentration of 100 mM maintained until the DNA extraction date. Plants watered with the same regime but with deionized water were used as controls. The seedlings were agro-inoculated nine days after sowing and total DNA extraction was done at 21 dpi. Physiological changes in plants caused by the high NaCl concentration were assessed by visual observations of darker green leaves and reduced root growth (Fig 1B), as well as through shoot size measurement (Fig A in S2 Appendix) at 21 dpi.

#### Different light intensities.

*V. faba* plants were grown in a growth chamber with 13h/11h day/night photoperiod, 25°C/18°C day/night temperature. Light was maintained at “standard intensity” until virus inoculation. Plantlets were agro-inoculated when nine days old and immediately placed under two different light intensities in the same growth chamber. The light intensity at the average height of the apices was measured after inoculation (0 dpi), at mid-growth (11 dpi) and DNA sampling time (19 dpi) using a spectrophotometer. Data is presented in Fig B in S2 Appendix. Physiological changes in plants induced by the different light intensities were determined by visual observations and shoot size measurement at 19 dpi (Fig 1C).

#### Different temperatures.

*V. faba* plantlets were grown in a growth chamber with 13h/11h day/night photoperiod and 25°C/18°C day/night temperature. When nine days-old, they were agro-inoculated and placed back into the same conditions to allow the transfer of the binary plasmids from agrobacteria clones to the plant. After two days, inoculated plants were transferred in a climatic chamber with the same photoperiod but 30°C/25°C day/night temperatures for the “higher temperatures” modality or 20°C/15°C day/night for the “cooler temperatures” modality, until DNA extraction. Total DNA extraction was carried out at 21 dpi for the 30°C/25°C day/night modality, and at 23 dpi and 28 dpi for the 20°C/15°C day/night modality in order to compensate for any delay in the infection stage resulting from a slower metabolism of plants at colder temperatures. Statistical analysis of FBNSV genome formula showed no significant differences between the two dates of sampling of the colder temperatures (23 or 28 dpi; Table B in S1 Appendix). Thus, we further use the data from the first sampling date (23 dpi) for comparison with the warmer temperature modality.

#### Co-infection with a potyvirus.

The bean yellow mosaic virus (BYMV) infectious clone used in the co-inoculation experiments was provided by E. Johansen, University of Copenhagen, Denmark [44,45]. Fifteen-day-old *V. faba* were mechanically inoculated with a crude extract of BYMV infected tissues in sodium phosphate buffer (0.04 M) added with carborundum powder. FBNSV was inoculated when the light green mosaic symptoms of BYMV were visible (four or seven dpi, depending on the plant set repeat). For this, two viruliferous aphids (*Acyrthosiphon pisum*, clone 210) that had acquired FBNSV during three days on infected plants were deposited on each BYMV-infected recipient plant. Plants not exposed to BYMV were inoculated with FBNSV in parallel to provide controls. After three days of inoculation access period, the aphids were eliminated by treatment with Pirimor G (1 g/L^-1^ in water). The plants were grown in a growth chamber with 13h/11h day/night photoperiod, 25°C/18°C day/night temperature. Total DNA was extracted from plants showing both FBNSV and BYMV symptoms (or only FBNSV symptoms for controls) 21 days after FBNSV inoculation. Physiological changes in plants caused by BYMV infection were determined by observing mosaic symptoms, typical of the disease (Fig 1D).

#### Aphid infestation.

*V. faba* were grown in a growth chamber with 13h/11h day/night photoperiod, 25°C/18°C day/night temperature. When nine-day old, the plantlets were agro-inoculated with FBNSV. The next day, ten non-viruliferous adult aphids (*Aphis craccivora*) were placed on each of them and the colony left to freely develop. Plants without aphids grown in parallel were used as control. At 14 dpi, the aphid colony had increased substantially, colonizing all plants and covering nearly the entire surface of their leaves and stem, to the point of threatening the survival of the plants. Total DNA was extracted and the impact of aphid overcrowding on plant physiology and development was ascertained by visual observations and shoot size measurement at 14 dpi (Fig 1E).

#### Nitrogen deficiency and rhizobial symbiosis.

*V. faba* seeds were surface-sterilized by soaking in 70% ethanol for 1 min, followed by a 10 min treatment in a 3.3% bleach solution containing Tween 20, and then rinsed four times with sterile water for 2 min each. Sterilized seedlings were soaked in water for 8h to induce germination and placed in Petri dishes containing 0.9% agar. After germination, seedlings were transplanted into vermiculite with Buffered Nodulation Medium (BNM [46],) in individual Magenta culture boxes and transferred to a greenhouse. At the same time, plants constituting the rhizobial symbiosis modality were inoculated by depositing 1 mL of *Rhizobium leguminosarum* strain RLV3841 (provided by E. Giraud, PHIM, Montpellier, France) culture (optical density (600 nm) = 1) on the vermiculite. Thirty-five days later, when the plants cultivated without symbiosis showed a phenotype of N deficiency, FBNSV was inoculated using 15 viruliferous aphids (*A. craccivora*). Total DNA was extracted from FBNSV-symptomatic plants 21 days after FBNSV inoculation. Physiological differences between plants grown under N deficiency and rhizobial symbiosis were assessed by visual observations, measurement of shoot size and fresh weight (Fig 1F, Fig C in S2 Appendix).

### DNA extraction and quantification

Total DNA was extracted from symptomatic apical leaves following the method described earlier [47]. Under water deprivation condition, however, the plants did not show any FBNSV symptoms, and so DNA had to be extracted from all plants. The concentration of the total DNA extracted was estimated using a spectrophotometer NanoDrop™ 2000 (Thermo Scientific™, Waltham, MA, USA). The concentration of each FBNSV segment was estimated by qPCR using a LightCycler 480 thermocycler (Roche, Indianapolis, Ind, USA) as previously described [48]. Plants that did not test positive for all eight FBNSV segments were excluded from the analysis.

### Data analysis

Statistical analyses on plant size/weight were done with ANOVA and Tukey HSD tests on non-transformed data, as normality and homoscedasticity were confirmed in all cases, using the package “stats” of the software RStudio (https://www.rstudio.com; Tables C and D in S1 Appendix).

Total FBNSV accumulation for each sample was obtained by adding the estimated concentrations of the eight segments and normalizing by the concentration of the total DNA extracted. Statistical analyses on virus load were done with ANOVA and Tukey HSD tests on log-transformed data to make their distribution closer to a normal distribution (Table E in S1 Appendix). Among all conditions, only residuals from the light intensity experiment data did not follow a normal distribution (p-value = 3.064e-05) and did not satisfy the homoscedasticity assumption after log-transformation (p-value = 0.01523). The data from this experiment were nevertheless processed using a parametric test for the sake of consistency with the other conditions tested.

The genome formula was calculated by expressing the concentration of each FBNSV segment relatively to the sum of the concentrations of all eight segments. Because the assumptions of normal distribution and/or homoscedasticity of residuals were not respected for most conditions even on logit-transformed data, comparisons of segment relative frequencies in the different conditions were performed with the non-parametric Scheirer Ray Hare and Dunn tests and Bonferroni correction on non-transformed data, using the packages “rcompanion” and “FSA” of the software RStudio (Table F in S1 Appendix). Our statistical analysis method differs from that used by Boezen *et al*. [49] based on PERMANOVA on the genome formula distance metric, considering the Euclidean distance between two values of the genome formula. The PERMANOVA allows for the simultaneous analysis of all segments using a single metric while being less sensitive to the parametric assumptions of the model (notably normal distribution) [50]. However, variations in genome formula can occur in different ways without necessarily altering the genome formula distance metric. This is for example the case when a decrease in the accumulation of one genome segment is compensated by an equivalent increase in another segment accumulation. For these reasons, we chose to use non-parametric ANOVA (Scheirer Ray Hare) tests on segment relative frequencies, which overcomes the problem of the assumptions in parametric ANOVA while being able to identify the segments responsible for potential genome formula changes. However, to reassure the reader that our inferences do not depend on the specific statistical method, we nevertheless carried out an analysis using the PERMANOVA technique on genome formula distances (Table G in S1 Appendix), which essentially yields the same results.

## Results

### Impact of growing conditions on plant and FBNSV infection phenotypes

The plant physiology changes caused by the different growing conditions were assessed at the time of DNA sampling by visual observation and measurement of the plant shoot size and/or fresh weight depending on what we judged most relevant to the growing condition tested. Fig 1 highlights phenotypic changes induced by water deficiency, high NaCl concentration, different light intensities, co-infection with an RNA virus (BYMV), aphid infestation and N deficiency or rhizobial symbiosis.

Plants grown under water deficiency showed the first signs of wilting from around 5 dpi and the wilting became increasingly severe until 19 dpi (Fig 1A, left panel). In order to facilitate DNA extraction from plants grown under water deficit, these were rehydrated the day before sampling. After one night of rehydration, the plants had recovered their turgor. They were smaller than the regularly watered control plants (Fig 1A right panel). Despite the undeniable presence of the virus (see next section), no symptoms of infection were visible before or after rehydration.

Plants watered with a NaCl solution had a similar size (Fig A in S2 Appendix), but they consistently showed a reduced root system (Fig 1B) and a slightly darker leaf color when compared to control plants watered with deionized water. Symptoms caused by FBNSV infection were similar to those of control plants (Fig 1B).

Plants grown under lower light intensity were etiolated, resulting in an increased size compared to plants grown under high light intensity (Fig 1C). The disease symptoms appeared milder in plants grown under high light intensity compared to those cultivated under low light intensity.

Phenotypic observation of plants grown under colder temperatures (20°C/15°C day/night) showed slower overall growth. The disease symptoms appeared perhaps milder in plants grown in cold versus warmer temperature, but this was only marginally noticeable. Because we did not notice striking morphological differences, beyond slower growth, this treatment is not illustrated in Fig 1.

Co-infections of *V. faba* with FBNSV and BYMV resulted in plant dwarfing and leaf curling, typical of FBNSV infection, together with a light green mosaic caused by BYMV infection (Fig 1D). Thus, co-infection results in the addition of symptoms of both diseases, with no noticeable synergism or antagonism between the two.

Aphid-infested plants had all leaves curled inwards, whereas only the apex leaves were curled in non-infested plants (Fig 1E, left panel). In addition, infested plants were smaller than plants grown without aphids (Fig 1E, right panel). Symptoms of viral infection might be similar in the two cases, but because aphid infestations produce pronounced inward leaf curling, the FBNSV symptoms were largely masked.

Finally, plants grown in symbiosis with *R. leguminosarum* showed a dark green color and an increased shoot fresh weight compared to plants grown without symbiosis which displayed a yellow color due to N deficiency (Fig 1F). Root weight was not statistically significantly affected (Fig C_C in S2 Appendix). Symptoms caused by FBNSV infection were similar for both symbiotic and non-symbiotic modalities (Fig 1F, left panel).

### Impact of the plant growing conditions on FBNSV within host fitness

**Fig 2:**
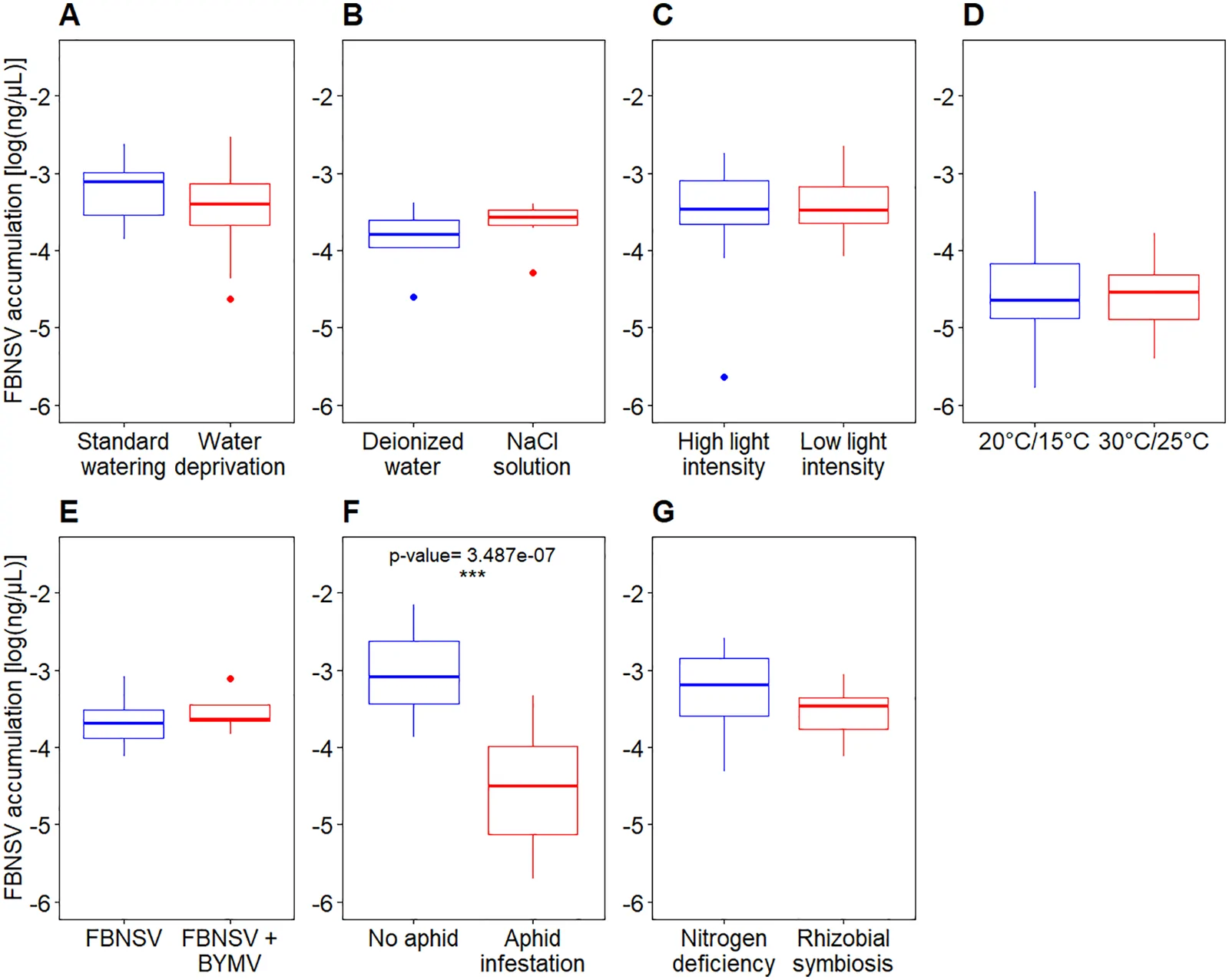
FBNSV accumulation in *V. faba* grown under different conditions. (A) water deprivation, (B) high NaCl concentration, (C) different light intensities, (D) different temperatures, (E) BYMV co-infections, (F) aphid infestation or (G) nitrogen deficiency and rhizobial symbiosis. FBNSV accumulation was obtained by adding the concentrations of the eight segments estimated by qPCR and normalizing by the concentration of the total DNA extracted. The central bar represents the median and the edges of the rectangle the first and third quartiles. The outer bars delineate 1.5 times the distance between the 25th and 75th percentile of the distribution. The dots represent the outliers. Statistically significant differences between conditions were assessed on log-transformed data based on ANOVA and Tukey HSD tests. A statistically significant difference was demonstrated solely in F with a p < 0.001 and is indicated by *** (also see Table E in S1 Appendix).

The severity of the symptoms caused by the FBNSV infection can be influenced by variations of the plant phenotype induced by the growing conditions, not necessarily reflecting any actual change in virus fitness. We thus used virus accumulation as an indicator of whether the within-host virus fitness is affected by the physiological state of the plant. Despite the reduction (light, temperature) or absence (water deprivation) of virus-induced symptoms for some conditions, the total concentration of FBNSV in plants was not affected by most growing conditions tested (Fig 2; Table E in S1 Appendix). Among all conditions, only the aphid infestation statistically significantly decreased the total concentration of FBNSV.

### FBNSV genome formula in plants grown under different conditions

**Fig 3:**
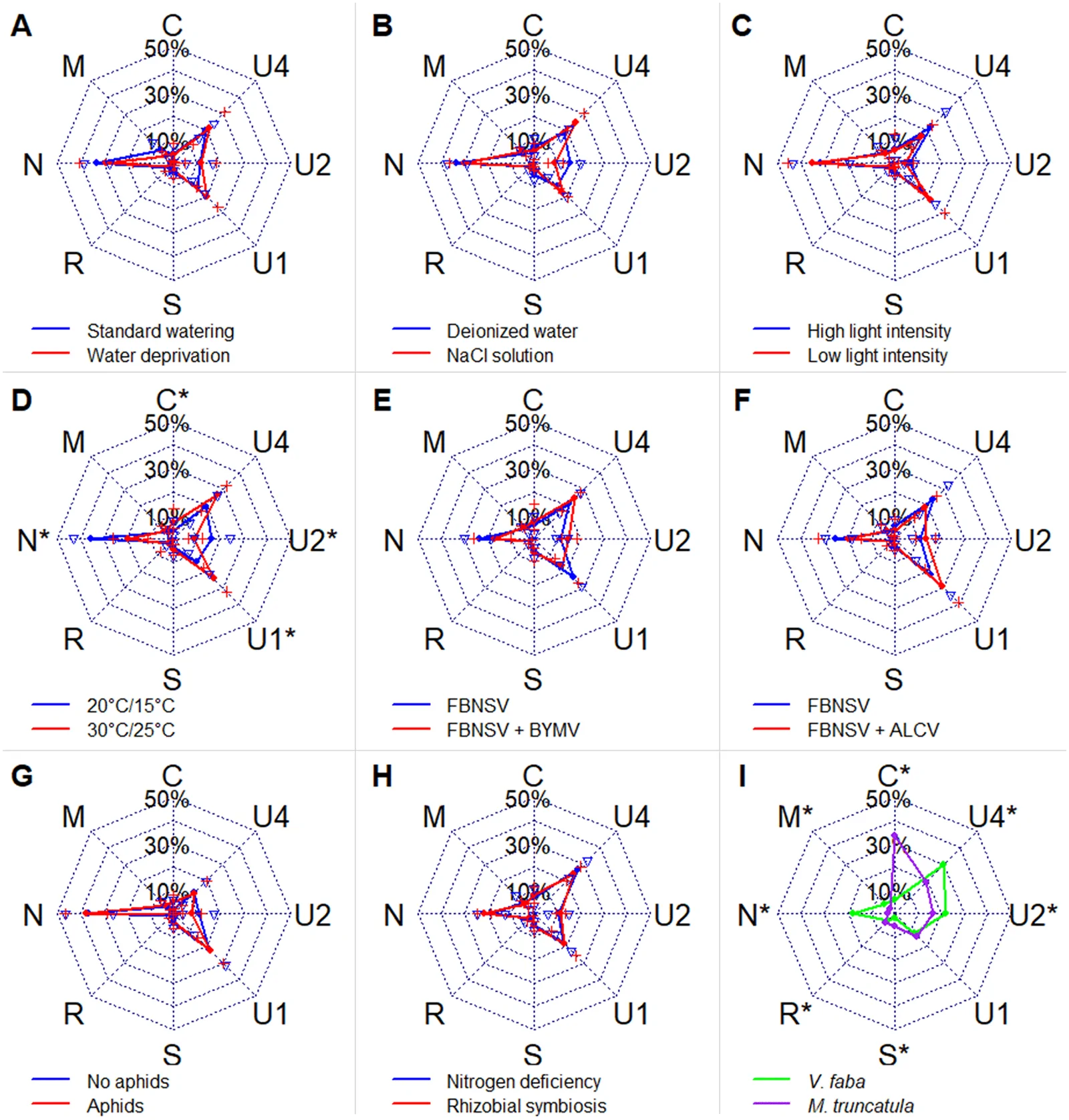
FBNSV genome formula in *V. faba* grown under different conditions. (A) water deprivation, (B) high NaCl concentration, (C) different light intensities, (D) different temperatures, (E) BYMV co-infections, (F) ALCV co-infections, (G) aphid infestation or (H) nitrogen deficiency and rhizobial symbiosis. FBNSV genome segments accumulation in the plants tested in Fig 2 was estimated by qPCR and the relative frequency of the segments was determined. Standard deviations are represented by blue triangles or red crosses. Asterisks associated to segment names indicate when the differences in frequencies between a given growing condition and its control plants are statistically significant (Scheirer Ray Hare (p ≤ 0.05) and post-hoc Dunn tests (Bonferroni correction) on non-transformed data). (I) Comparison of FBNSV genome formula in *V. faba* and *M. truncatula*. This panel is adapted from Sicard *et al*., 2013, for comparison, to simply remind how genome formula can be affected by the plant genotype.

Genome formulas in plants grown under the different conditions tested were established by determining the frequency of each FBNSV genome segment within the total virus population measured by qPCR (Fig 3). To cover as many pathways that may interfere with FBNSV infection as possible, we also included data generated in an earlier study on co-infection of FBNSV with the ssDNA virus ALCV [47] in our analysis. In that study, it was shown that co-infection with ALCV does not interfere with the symptoms caused by FBNSV and does not impact total FBNSV accumulation [47].

Overall, the genome formula was not statistically significantly affected by most growing conditions tested, but only by temperature (Fig 3, Table F in S1 Appendix). FBNSV populations infecting plants grown under colder temperatures had lower relative frequencies for segments C and U1 and higher for segments N and U2 compared to those infecting plants grown under higher temperatures (Fig 3D). Despite being statistically significant, these variations remain limited. They do not drastically modify the pattern of accumulation of the FBNSV genome segments, in contrast to what is observed during a host change (Fig 3I).

## Discussion

To our best knowledge, the effect of an environmental condition other than the host genotype on the genome formula has been evaluated in only one instance, that of coinfection of cucumber mosaic virus (CMV; family *Bromoviridae*, genus *Cucumovirus*) with either alfalfa mosaic virus (AMV; family *Bromoviridae*, genus *Alfamovirus*), potato virus Y (PVY; family *Potyviridae*, genus *Potyvirus*), or both [49]. In this case, it was found that coinfection with AMV, but not with PVY or both, impacted the CMV genome formula by affecting its dispersion. In our study, even when using the statistical approach of Boezen *et al.,* (Tables F and G in S1 Appendix), neither the co-infection with a potyvirus, nor that with a geminivirus were found to impact the genome formula. It is difficult to interpret this discrepancy as several aspects, methodological (sequential infections in our case *vs*. simultaneous coinfection in Boezen *et al*.,) and biological (no effect of coinfection on virus accumulation in our case *vs.* reduction of CMV accumulation under coinfection in Boezen *et al.,*), differ between the studies beyond the identity of the viruses involved. Importantly, the Bromoviridae have a positive-sense single-stranded RNA ((+)ssRNA) genome. Consequently, as acknowledged by Boezen et al., in the ‘Transcriptome vs. genome formula’ section of their paper, in (+)ssRNA viruses we do not know to what extent what is quantified reflects the relative accumulation of transcripts vs. the relative accumulation of genome segments. Because of this, direct comparisons with our results are difficult, and perhaps the difference in the outcomes that we observe reflects simply this. Setting this issue aside, and more generally, the extent to which our results should extend to other types of viruses should depend mostly on how various types of viruses modulate their gene expression.

To better appreciate the observations reported in this manuscript, it is to be reminded that, when switching host species, the FBNSV genome formula can undergo massive changes. Indeed, in the example of FBNSV transmission from *V. faba* to *M. truncatula*, the relative frequency of almost all segments, except U1, was statistically significantly impacted ([11], Fig 3). More importantly, the overall frequency pattern was drastically altered, with some segments that were very frequent in the first host becoming rare in the second, and *vice versa* [11]. For example, segment C represented 6% of the viral population in *V. faba* and increased to 33% when the infection was transferred to *M. truncatula* ([11], Fig 3). When compared to the situation earlier described [11,12] and summarized above, all growing conditions tested in our study have a much lower impact on the FBNSV genome formula (Fig 3). For most conditions tested, some small and not statistically significant variations are observed for some segments depending on the growing condition. The only statistically significant differences were found for different growing temperatures but, even in this case, the general pattern of the formula is not qualitatively affected.

As opposed to all other growing conditions, under aphid infestation, the total FBNSV accumulation was significantly reduced (Fig 2). The decrease in viral load indicates that the FBNSV infection is altered by this plant condition, but apparently with no impact on the genome formula. The reduction in FBNSV accumulation may be due to the plant’s defenses against aphids, which probably also act on virus infection. Specifically, aphid feeding induces local cytoskeletal rearrangement and callose deposition contributing to clogging the punctured phloem sieve tubes [51,52], which may hinder virus propagation. The clogging reorganizes phloem sap flow, which would then be redirected through lateral connections between parallel sieve tubes [53,54], making certain regions of the phloem inaccessible to the virus. Thus, the observed decrease in total FBNSV accumulation would result from a smaller number of infection foci, while the replication dynamics at each replication site remain unchanged and do not impact the genome formula.

For all other conditions tested, the total FBNSV accumulation was not different from that in plants grown under standard conditions (Fig 2, Table E in S1 Appendix, [47]), suggesting that FBNSV within-host fitness is not significantly affected by the corresponding physiological status of the plant. That we in addition detected only very small modifications of the genome formula can be interpreted in different ways: (i) the tested physiological conditions of the plant do not interfere in any way with the viral infection; (ii) small genome formula variations, statistically undetectable by our sample sizes, are sufficient to adjust the virus population accumulation to physiological changes in the host; (iii) in this panel of growing conditions, the virus adjustments to the plant physiology do not proceed through genome formula modifications.

(i) The physiological condition of the plant may not interfere with FBNSV infection. This hypothesis cannot be excluded but appears poorly satisfactory. It has been shown that environmental constraints can have a direct impact on virus fitness and epidemiological parameters [55]. Interferences due to crosstalk between abiotic and biotic stress (including viruses) response pathways have been evidenced [56]. More specifically, in the family *Geminiviridae*, the tomato yellow leaf curl virus (TYLCV) confers enhanced tolerance to drought [57,58] or high temperatures [59]. *Geminiviridae* and *Nanoviridae* sharing some genomic similarities and protein analogies [20,29,60,61], a complete absence of crosstalk with abiotic stress pathways such as those mobilized under drought or heat would be surprising. In addition, interactions between geminivirus proteins and host proteins involved in stress response processes have been identified. For example, the heat shock protein HSP70 which is involved in plant responses to heat and saline stresses, is also implicated in the translocation of the tomato yellow leaf curl Sardinia virus (TYLSV) coat protein (CP) from the cytoplasm to the nucleus of the host cell [59,62]. The movement protein (MP) of the cabbage leaf curl virus (CaLCuV) interacts with the *Arabidopsis thaliana* synaptotagmin SYTA [63], which has consistently been shown to impact on cell-to-cell movement of various viruses *via* plasmodesmata [64]. SYTA belongs to the synaptotagmin family that are Ca^2+^/lipid binding proteins including Ca^2+^ sensors and is required for the maintenance of plasma membrane integrity and for tolerance to saline stress in *A. thaliana* [65,66]. Changes in the accumulation or availability of such proteins under stress conditions could force the virus to modify its genes expression to compensate for these modifications. In nanoviruses, it has been shown that the nuclear shuttle protein (NSP) encoded by segment N of the pea necrotic yellow dwarf virus (PNYDV) interacts with the host Ras-GAP SH3-domain–binding protein (G3BP) [60]. G3BP is a protein involved in the formation of stress granules, which are cellular structures that regulate protein expression during the stress response. NSP of FBNSV appears mandatory for aphid transmission but has no identified function in plants. The absence of the segment N has no effect on FBNSV infection of *V. faba*, nor on its genome formula, under standard laboratory growing conditions [48,67]. One could have expected a role of NSP *in planta* specifically under stress conditions, but in no case did we observe large changes in the frequency of the corresponding segment N. Besides the interactions between viral and host proteins, changes in cellular parameters generated by different culture conditions could have an impact on the production, stability or functionality of viral proteins. An increase in the copy number of the corresponding genes could compensate for this loss of functionality. For all these reasons, it seems unlikely that the physiological state of the plant does not interfere with FBNSV infection. To find out whether the virus adjusts its gene expression to conditions generated by physiological changes in the plant, viral mRNAs amount and translation should be quantified in the future. If the amount of FBNSV gene expression products remains unchanged, this would support the hypothesis that the stress conditions tested here do not interfere with viral infection, and therefore do not constrain the virus to adapt by modulating the genome formula or through any other mechanism of gene expression regulation. The opposite situation, i.e., plant stressors lead to a modification of the relative abundance of viral expression products, would indicate that the FBNSV infection is affected by the plant’s physiological condition. In that case, hypotheses (ii) and (iii) would be favored.
(ii) Small variations may be sufficient to compensate for changes of the host physiological status. In the different conditions tested, we observed slight variations in the frequency of certain segments, which could somewhat be masked for some other segments by the inter-plant variability. Thus, we can speculate that small changes in gene copy number could have large effects (as predicted earlier [68]) and be sufficient to adjust viral gene expression to a slightly modified environment. This contrasts with the drastic changes in the FBNSV genome formula between two host plants (Fig 3I, [11,12]). It may be hypothesized that functional requirements differ and that certain viral proteins interact with different cellular factors depending on the host genotype, which would thus induce qualitative differences in the virus’ environment. This would require major changes in the expression of different viral genes from one host to another, and thus large variations in their copy number. Conversely, changes in physiological state could only represent quantitative changes, for example altering the amount of host proteins available, and small variations in the copy number of viral genes would suffice to rapidly adjust.
(iii) Finally, adjustments in FBNSV gene expression may not involve variations of the genome formula, but other mechanisms. Because of their high mutation rates, large population sizes and short generation times, viruses can adapt rapidly to adverse environments [55]. Nanoviruses have particularly high mutation rates [69], potentially enabling rapid virus adaptation by selecting the variants best suited to new cellular conditions. However, the recent demonstration [12] that no mutations are responsible for the genome formula modification when the FBNSV switches from *V. faba* to *M. truncatula* renders this hypothesis for immediate adaptation to stress conditions unlikely. An alternative possibility is the modification of gene expression through transcriptional and/or post-transcriptional regulation mechanisms under stress conditions, conferring a plasticity of the regulation of gene expression at different levels.

Whatever the explanation for the very limited variations observed here, altering plant physiology is clearly not reshaping the FBNSV genome formula in the way host switching does. The variation in genome formula typically observed across individual hosts of the same species in any experiment, and potentially even more in field studies, could in principle be ascribed to uncontrolled variation in the microenvironmental conditions in which individual hosts grow. If this was the case such variation would not be due to ‘noise’, but to unaccounted for variation in factors with deterministic effects. Our results strongly suggest that this is unlikely to be the case, since controlling for such variation in host physiology does not have qualitatively noticeable consequences on the genome formula. Therefore, at least under our current understanding, interindividual variation in genome formula is better ascribed to noise at some stage of the infection process.

More generally, the results presented here suggest that genome formula remodeling is not a systematic response, and may instead respond mainly to more profound changes in the viral cellular environment. Therefore, the question is no longer whether the genome formula is plastic, but which environmental changes are actually strong or specific enough to modify it.

## Supporting information

S1 Appendix Table A. Statistical analysis of the impact of the one-day rehydration of infected plants on the FBNSV segment relative frequencies. Comparison of segment relative frequencies was assessed through Scheirer Ray Hare tests (package “rcompanion”). We provide the output of a full model: frequency = segment * modality, where modality corresponds to one-day rehydration or no rehydration. The p-value indicating statistically significant differences (p ≤ 0.05) is in red. A statistically significant “segment” effect indicates that the different segments accumulate at different relative frequencies, *i.e.,* that the genome formula results in an uneven distribution of segment relative frequencies. A statistically significant “segment * modality” interaction indicates that the genome formula is statistically significantly modified by the modality. No statistically significant effect of the one-day rehydration was found. **Table B**. Statistical analysis of the impact of the date of sampling on the FBNSV segment relative frequencies in plants grown under 20/15°C day/night temperatures. Comparison of segment relative frequencies was assessed through Scheirer Ray Hare tests (package “rcompanion”). We provide the output of a full model frequency = segment * modality where modality corresponds to the date of sampling. The p-value indicating statistically significant differences (p ≤ 0.05) is in red. A statistically significant “segment” effect indicates that the different segments accumulate at different relative frequencies, *i.e.,* that the genome formula results in an uneven distribution of segment relative frequencies. A statistically significant “segment * modality” interaction would indicate that the genome formula is statistically significantly modified by the modality. No statistically significant effect of date of sampling was found. **Table C.** Statistical analysis on size of *V. faba* plants grown in different specific conditions compared to that in plants grown in reference conditions. Comparisons were assessed through ANOVA and Tukey HSD tests on non-transformed data using RStudio (package “stats”). df indicates the number of observations and F-value the statistic value. The p-value indicating statistically significant difference (p ≤ 0.05) is in red. **Table D.** Statistical analysis on weight of *V. faba* plants grown in nitrogen deficiency compared to that of plants grown in symbiosis with R. *leguminosarum*. Comparisons were assessed through ANOVA and Tukey HSD tests on non-transformed data using RStudio (package “stats”). df indicates the number of observations and F-value the statistic value. The p-value indicating statistically significant difference (p ≤ 0.05) is in red. **Table E.** Statistical analysis of FBNSV accumulation in *V. faba* plants grown in different specific conditions compared to that in plants grown in reference conditions. Comparisons of FBNSV accumulation were assessed through ANOVA and Tukey HSD tests on Log-transformed data using RStudio (package “stats”). df indicates the number of observations and F-value the statistic value. The p-value indicating statistically significant difference (p ≤ 0.05) is in red. **Table F.** Statistical analysis of the relative frequencies of each FBNSV segment in *V. faba* plants grown in different specific conditions compared to the genome formula in plants grown in reference conditions. Comparisons of segment relative frequencies were assessed through Scheirer Ray Hare and Dunn tests (packages “rcompanion” and “FSA”). For each growing condition tested, we first provide the output of a full model frequency = segment * modality where modality corresponds to the growth condition. A statistically significant “segment” effect indicates that the different segments accumulate at different relative frequencies, *i.e.,* that the genome formula results in an uneven distribution of segment relative frequencies. A statistically significant “segment * modality” interaction indicates that the genome formula is statistically significantly modified by the modality. Statistically significant difference was found for plants grown under different temperatures only. For this condition, we provide the output of per segment comparisons of the modality to identify which segments’ frequencies are significantly affected. Segments with a positive Z had higher relative frequency under cold relative to high temperatures. The p-values indicating statistically significant differences after Bonferroni correction (p ≤ 0.05) are in red. **Table G.** Statistical analysis of the distance of FBNSV genome formula in *V. faba* plants grown in different specific conditions compared to the genome formula in plants grown in reference conditions. The analysis was performed based on the method used by Boezen *et al*. (49). Pairwise distances between the genome formula of the tested growing condition and the genome formula of the reference condition were computed using the vegdist function with Euclidean distance. The significance of these distances was assessed using PERMANOVA on the distance matrix, using the adonis2 function. Statistically significant differences were found for the high NaCl concentration and the different temperatures conditions. Multivariate dispersion of genome formula was compared using PERMDISP2 (betadisper, permutest) with 10⁴ permutations. In almost all cases, multivariate dispersion did not statistically differ between the tested growing condition and its reference. The only exception was the water deprivation condition, for which we provide the mean distance of the genome formulas. The functions vegdist, adonis2, betadisper, and permutest are part of the package “vegan” of RStudio. The p-value indicating statistically significant difference (p ≤ 0.05) is in red. Thus this analysis agrees with the non-parametric ANOVA analysis we present in the main text in identifying a statistically significant effect of temperature on genome formula, and adds a small statistically significant effect of high NaCl concentration and an effect of water deprivation on the variability of the genome formula, but not on the mean genome formula distance.
(DOCX)

S2 Appendix Fig A. Shoot size of FBNSV-infected *V. faba* watered with deionized water (blue) or NaCl solution (red). Plant shoot size was measured before sampling at 21 dpi. In boxplots, the central bar represents the median and the edges of the rectangle the first and third quartiles. The outer bars delineate 1.5 times the distance between the 25^th^ and 75^th^ percentile of the distribution. The dots represent the outliers. No statistically significant difference was found between the two conditions based on ANOVA test on non-transformed data (p-value = 0.085; also see Table C in S1 Appendix). **Fig B.** Light intensity measured at the height of FBNSV-infected *V. faba*. Light was maintained at “standard intensity” (green) until virus inoculation. After agro-inoculation, plantlets were placed under “higher light intensity” (blue) or “low light intensity” (red) in the same growth chamber. The light intensity (Photosynthetic Photon Flux Density; PPFD) at plant height was measured after inoculation (0 dpi), at mid-growth (11 dpi) and DNA sampling time (19 dpi) using a spectrophotometer. The plant height corresponds to the average height of the plants for each light intensity condition (0 dpi: 6 cm; 11dpi: 23.11 cm for high light intensity, 30.60 cm for low light intensity; 19 dpi: 32.71 cm for high light intensity and 39.57 cm for low light intensity). In boxplots, the central bar represents the median of the five measurement points (at the center and four sides of the shelf) and the edges of the rectangle the first and third quartiles. The outer bars delineate 1.5 times the distance between the 25^th^ and 75^th^ percentile of the distribution. **Fig C.** Shoot size (A), total fresh weight (B) and root fresh weight (C) of FBNSV-infected *V. faba* grown under nitrogen deficiency (blue) or rhizobial symbiosis (red). Plant shoot size (A), shoot fresh weight (Fig 1F), total fresh weight (B) and root fresh weight (C) were determined before DNA extraction, 21 days after FBNSV inoculation. Total fresh weight was obtained by adding the shoot and root weights. In boxplots, the central bar represents the median and the edges of the rectangle the first and third quartiles. The outer bars delineate 1.5 times the distance between the 25^th^ and 75^th^ percentile of the distribution. The dots represent the outliers. Statistically significant differences between conditions were assessed on non-transformed data based on ANOVA (p ≤ 0.05). While differences in total fresh (B) weight and shoot fresh weight (Fig 1F) could be observed, no statistically significant difference was found for shoot size (A; p-value = 0.4507) and root fresh weight (C; p-value = 0.1745) (also see Tables C and D in S1 Appendix).
(DOCX)
