# Supplementary material for: Small impact of the host plant physiological state on the genome formula of a nanovirus
Source: PLoS Pathog. 2026 Sep 21;22(9):e1014641. doi: 10.1371/journal.ppat.1014641 (PMC13614657; doi:10.1371/journal.ppat.1014641)
Supplement: Supplementary file 1 [file ppat.1014641.s001.docx]

**Table A. Statistical analysis of the impact of the one-day rehydration of infected plants on the FBNSV segments relative frequency.** Comparison of segments relative frequencies was assessed through Scheirer Ray Hare tests (package “rcompanion”). We provide the output of a full model: frequency = segment * modality, where modality corresponds to one-day rehydration or no rehydration. The p-value indicating statistically significant differences (p≤0.05) is in red. A statistically significant “segment” effect indicates that the different segments accumulate at different relative frequencies, *i.e.* that the genome formula results in an uneven distribution of segment relative frequencies. A statistically significant “segment * modality” interaction indicates that the genome formula is statistically significantly modified by the modality. No statistically significant effect of the one-day rehydration was found.

| **Source** | **DF** | **Sum of Squares** | **H** | **p-value** |
| --- | --- | --- | --- | --- |
| segment | 7 | 644538 | 153.461 | <10^-5^ |
| modality | 1 | 901 | 0.214 | 0.64332 |
| segment*modality | 7 | 5388 | 1.283 | 0.98889 |
| residuals | 208 | 285774 |  |  |

**Table B. Statistical analysis of the impact of the date of sampling on the FBNSV segments relative frequency in plants grown under 20/15°C day/night temperatures.** Comparison of segment relative frequencies was assessed through Scheirer Ray Hare tests (package “rcompanion”). We provide the output of a full model frequency = segment * modality where modality corresponds to the date of sampling. The p-value indicating statistically significant differences (p≤0.05) is in red. A statistically significant “segment” effect indicates that the different segments accumulate at different relative frequencies, *i.e.* that the genome formula results in an uneven distribution of segment relative frequencies. A statistically significant “segment * modality” interaction would indicate that the genome formula is statistically significantly modified by the modality. No statistically significant effect of date of sampling was found.

| **Source** | **DF** | **Sum of Squares** | **H** | **p-value** |
| --- | --- | --- | --- | --- |
| segment | 7 | 1692129 | 207.929 | <10^-5^ |
| modality | 1 | 2317 | 0.285 | 0.59361 |
| segment*modality | 7 | 18046 | 2.217 | 0.94683 |
| residuals | 296 | 818426 |  |  |

**Table C. Statistical analysis on size of *V. faba* plants grown in different specific conditions compared to that in plants grown in reference conditions.** Comparisons were assessed through ANOVA and Tukey HSD tests on non-transformed data using RStudio (package “stats”). df indicates the number of observations and F-value the statistic value. The p-value indicating statistically significant difference (p≤0.05) is in red.

|  | **Water** | **NaCl concentration** | **Light intensity** | **Aphid infestation** | **Nitrogen and symbiosis** |
| --- | --- | --- | --- | --- | --- |
| **df** | 1 | 1 | 1 | 1 | 1 |
| **F-value** | 53.056 | 3.252 | 16.2 | 30.71 | 0.59 |
| **p-value** | 3.393e-08 | 0.085 | 0.000182 | 7.65e-07 | 0.451 |

**Table D. Statistical analysis on weight of *V. faba* plants grown in nitrogen deficiency compared to that of plants grown in symbiosis with R. *leguminosarum*.** Comparisons were assessed through ANOVA and Tukey HSD tests on non-transformed data using RStudio (package “stats”). df indicates the number of observations and F-value the statistic value. The p-value indicating statistically significant difference (p≤0.05) is in red.

|  | **Shoot** | **Roots** | **Total** |
| --- | --- | --- | --- |
| **df** | 1 | 1 | 1 |
| **F-value** | 18.8 | 1.9686 | 13.48 |
| **p-value** | 0.000266 | 0.175 | 0.00134 |

**Table E. Statistical analysis of FBNSV accumulation in *V. faba* plants grown in different specific conditions compared to that in plants grown in reference conditions.** Comparisons of FBNSV accumulation were assessed through ANOVA and Tukey HSD tests on Log-transformed data using RStudio (package “stats”). df indicates the number of observations and F-value the statistic value. The p-value indicating statistically significant difference (p≤0.05) is in red.

|  | **Water** | **NaCl concentration** | **Light intensity** | **Temperatures** | **BYMV co-infection** | **Aphid infestation** | **Nitrogen and symbiosis** |
| --- | --- | --- | --- | --- | --- | --- | --- |
| **df** | 42 | 12 | 54 | 44 | 23 | 31 | 20 |
| **F-value** | 1.124 | 1.071 | 0.587 | 0.006 | 0.721 | 41.53 | 1.578 |
| **p-value** | 0.295 | 0.321 | 0.447 | 0.94 | 0.405 | 3.49e-07 | 0.224 |

**Table F. Statistical analysis of the relative frequencies of each FBNSV segment in *V. faba* plants grown in different specific conditions compared to the genome formula in plants grown in reference conditions.** Comparisons of segment relative frequencies were assessed through Scheirer Ray Hare and Dunn tests (packages “rcompanion” and “FSA”). For each growing condition tested, we first provide the output of a full model frequency = segment * modality where modality corresponds to the growth condition. A statistically significant “segment” effect indicates that the different segments accumulate at different relative frequencies, *i.e.* that the genome formula results in an uneven distribution of segment relative frequencies. A statistically significant “segment * modality” interaction indicates that the genome formula is statistically significantly modified by the modality. Statistically significant difference was found for plants grown under different temperatures only. For this condition, we provide the output of per segment comparisons of the modality to identify which segments’ frequency are significantly affected. Segments with a positive Z had higher relative frequency under cold relative to high temperatures. The p-values indicating statistically significant differences after Bonferroni correction (p≤0.05) are in red.

Water deprivation

Full model:

| **Source** | **DF** | **Sum of Squares** | **H** | **p-value** |
| --- | --- | --- | --- | --- |
| segment | 7 | 2771719 | 255.930 | <10^-5^ |
| modality | 1 | 9807 | 0.906 | 0.34129 |
| segment*modality | 7 | 45986 | 4.246 | 0.75102 |
| residuals | 344 | 1060457 |  |  |

High NaCl concentration

Full model:

| **Source** | **DF** | **Sum of Squares** | **H** | **p-value** |
| --- | --- | --- | --- | --- |
| segment | 7 | 151357 | 109.998 | <10^-5^ |
| modality | 1 | 488 | 0.355 | 0.55144 |
| segment*modality | 7 | 1016 | 2.918 | 0.89245 |
| residuals | 112 | 18891 |  |  |

Different light intensities

Full model:

| **Source** | **DF** | **Sum of Squares** | **H** | **p-value** |
| --- | --- | --- | --- | --- |
| segment | 7 | 4991609 | 308.696 | <10^-5^ |
| modality | 1 | 7425 | 0.459 | 0.49801 |
| segment*modality | 7 | 65540 | 4.053 | 0.77363 |
| residuals | 424 | 2034055 |  |  |

Different temperatures

Full model:

| **Source** | **DF** | **Sum of Squares** | **H** | **p-value** |
| --- | --- | --- | --- | --- |
| segment | 7 | 2846210 | 251.521 | <10^-6^ |
| modality | 1 | 21470 | 1.897 | 0.168384 |
| segment*modality | 7 | 259867 | 22.965 | 0.001729 |
| residuals | 352 | 1025426 |  |  |

Per segment comparisons:

| **Segment** | **Z** | **p-value unadjusted** | **p-value adjusted** |
| --- | --- | --- | --- |
| C | -3.402145 | 0.0006685909 | 5.348727e-03 |
| M | -1.728959 | 0.08381642 | 6.705314e-01 |
| N | 4.85224 | 1.220748e-06 | 9.765984e-06 |
| R | -1.528177 | 0.1264687 | 1000000e+00 |
| S | -0.7250474 | 0.468423 | 1000000e+00 |
| U1 | -4.116038 | 3.854407e-05 | 3.083526e-04 |
| U2 | 4.093729 | 4.244906e-05 | 3.395925e-04 |
| U4 | -2.398234 | 0.01647436 | 1.317949e-01 |

BYMV co-infections

Full model:

| **Source** | **DF** | **Sum of Squares** | **H** | **p-value** |
| --- | --- | --- | --- | --- |
| segment | 7 | 502126 | 149.888 | <10^-5^ |
| modality | 1 | 695 | 0.208 | 0.64868 |
| segment*modality | 7 | 12135 | 3.623 | 0.82208 |
| residuals | 184 | 151693 |  |  |

ALCV co-infections

Full model:

| **Source** | **DF** | **Sum of Squares** | **H** | **p-value** |
| --- | --- | --- | --- | --- |
| segment | 7 | 3310885 | 292.584 | <10^-5^ |
| modality | 1 | 1 | 0.000 | 0.99358 |
| segment*modality | 7 | 18814 | 1.663 | 0.97614 |
| residuals | 352 | 823272 |  |  |

Aphid infestation

Full model:

| **Source** | **DF** | **Sum of Squares** | **H** | **p-value** |
| --- | --- | --- | --- | --- |
| segment | 7 | 6862802 | 356.69 | <10^-5^ |
| modality | 1 | 652 | 0.03 | 0.85394 |
| segment*modality | 7 | 109168 | 5.67 | 0.57828 |
| residuals | 464 | 2243337 |  |  |

Nitrogen deficiency and rhizobial symbiosis

Full model:

| **Source** | **DF** | **Sum of Squares** | **H** | **p-value** |
| --- | --- | --- | --- | --- |
| segment | 7 | 361342 | 139.192 | <10^-5^ |
| modality | 1 | 3 | 0.001 | 0.97404 |
| segment*modality | 7 | 4208 | 1.621 | 0.97783 |
| residuals | 160 | 88747 |  |  |

**Table G. Statistical analysis of the distance of FBNSV genome formula in *V. faba* plants grown in different specific conditions compared to the genome formula in plants grown in reference conditions.** The analysis was performed based on the method used by Boezen et al. (49). Pairwise distances between the genome formula of the tested growing condition and the genome formula of the reference condition were computed using the vegdist function with Euclidean distance. The statistical significance of these distances was assessed using PERMANOVA on the distance matrix, using the adonis2 function. Statistically significant differences were found for the high NaCl concentration and the different temperatures conditions. Multivariate dispersion of genome formula was compared using PERMDISP2 (betadisper, permutest) with 10⁴ permutations. In almost all cases, multivariate dispersion did not statistically differ between the tested growing condition and its reference. The only exception was the water deprivation condition, for which we provide the mean distance of the genome formulas. The functions vegdist, adonis2, betadisper, and permutest are part of the package “vegan” of RStudio. The p-value indicating statistically significant difference (p ≤ 0.05) is in red. Thus, this analysis agrees with the non-parametric ANOVA analysis we present in the main text in identifying a statistically significant effect of temperature on genome formula, and adds a small statistically significant effect of high NaCl concentration and an effect of water deprivation on the variability of the genome formula, but not on the mean genome formula distance.

Water deprivation

PERMANOVA:

| **Source** | **DF** | **Sum of Squares** | **R2** | **F** | **p-value** |
| --- | --- | --- | --- | --- | --- |
| modality | 1 | 0.06451 | 0.05108 | 2.3147 | 0.05619 |
| residuals | 43 | 1.19843 | 0.94892 |  |  |
| total | 44 | 1.26294 | 1.00000 |  |  |

PERMDISP2:

| **Source** | **DF** | **Sum of Squares** | **Mean of Squares** | **F** | **p-value** |
| --- | --- | --- | --- | --- | --- |
| groups | 1 | 0.042642 | 0.042642 | 6.8542 | 0. 0115 |
| residual | 43 | 0.267513 | 0.006221 |  |  |

Mean distance of the genome formulas:

| **Modality** | **Standard watering** | **Water deprivation** |
| --- | --- | --- |
| **Mean distance** | 0.1024329 | 0.1659251 |

High NaCl concentration

PERMANOVA:

| **Source** | **DF** | **Sum of Squares** | **R2** | **F** | **p-value** |
| --- | --- | --- | --- | --- | --- |
| modality | 1 | 0.026686 | 0.1542 | 2.5523 | 0.0295 |
| residuals | 14 | 0.146379 | 0.8458 |  |  |
| total | 15 | 0.173065 | 1.0000 |  |  |

PERMDISP2:

| **Source** | **DF** | **Sum of Squares** | **Mean of Squares** | **F** | **p-value** |
| --- | --- | --- | --- | --- | --- |
| groups | 1 | 0.0000264 | 0.00002636 | 0.0138 | 0.925 |
| residual | 14 | 0.0267837 | 0.00191312 |  |  |

Different light intensities

PERMANOVA:

| **Source** | **DF** | **Sum of Squares** | **R2** | **F** | **p-value** |
| --- | --- | --- | --- | --- | --- |
| modality | 1 | 0.06235 | 0.03575 | 1.9651 | 0.1116 |
| residuals | 53 | 1.68173 | 0.96425 |  |  |
| total | 54 | 1.744081 | 1.00000 |  |  |

PERMDISP2:

| **Source** | **DF** | **Sum of Squares** | **Mean of Squares** | **F** | **p-value** |
| --- | --- | --- | --- | --- | --- |
| groups | 1 | 0.00434 | 0.0043423 | 0.4403 | 0.518 |
| residual | 53 | 0.52265 | 0.0098614 |  |  |

Different temperatures

PERMANOVA:

| **Source** | **DF** | **Sum of Squares** | **R2** | **H** | **p-value** |
| --- | --- | --- | --- | --- | --- |
| modality | 1 | 0.51429 | 0.33239 | 21.907 | 9.999e-05 |
| residuals | 44 | 1.03295 | 0.66761 |  |  |
| total | 45 | 1.54724 | 1.00000 |  |  |

PERMDISP2:

| **Source** | **DF** | **Sum of Squares** | **Mean of Squares** | **F** | **p-value** |
| --- | --- | --- | --- | --- | --- |
| groups | 1 | 0.000236 | 0.0002363 | 0.0618 | 0.8038 |
| residual | 44 | 0.168126 | 0.0038210 |  |  |

BYMV co-infections

PERMANOVA:

| **Source** | **DF** | **Sum of Squares** | **R2** | **F** | **p-value** |
| --- | --- | --- | --- | --- | --- |
| modality | 1 | 0.03025 | 0.0662 | 1.6306 | 0.1625 |
| residuals | 23 | 0.42669 | 0.9338 |  |  |
| total | 24 | 0.45694 | 1.0000 |  |  |

PERMDISP2:

| **Source** | **DF** | **Sum of Squares** | **Mean of Squares** | **F** | **p-value** |
| --- | --- | --- | --- | --- | --- |
| groups | 1 | 0.000030 | 0.00002969 | 0.0099 | 0.9208 |
| residual | 23 | 0.069188 | 0.00300816 |  |  |

ALCV co-infections

PERMANOVA:

| **Source** | **DF** | **Sum of Squares** | **R2** | **F** | **p-value** |
| --- | --- | --- | --- | --- | --- |
| modality | 1 | 0.4251 | 0.03313 | 1.5126 | 0.1996 |
| residuals | 44 | 1.23646 | 0.96677 |  |  |
| total | 45 | 1.27896 | 1.00000 |  |  |

PERMDISP2:

| **Source** | **DF** | **Sum of Squares** | **Mean of Squares** | **F** | **p-value** |
| --- | --- | --- | --- | --- | --- |
| groups | 1 | 0.000056 | 0.0000562 | 0.0099 | 0.9206 |
| residual | 44 | 0.250379 | 0.0056904 |  |  |

Aphid infestation

PERMANOVA:

| **Source** | **DF** | **Sum of Squares** | **R2** | **F** | **p-value** |
| --- | --- | --- | --- | --- | --- |
| modality | 1 | 0.05259 | 0.03226 | 1.9336 | 0.116 |
| residuals | 58 | 1.57751 | 0.96774 |  |  |
| total | 59 | 1.63010 | 1.00000 |  |  |

PERMDISP2:

| **Source** | **DF** | **Sum of Squares** | **Mean of Squares** | **F** | **p-value** |
| --- | --- | --- | --- | --- | --- |
| groups | 1 | 0.008437 | 0.0084366 | 1.8582 | 0.1766 |
| residual | 58 | 0.263337 | 0.0045403 |  |  |

Nitrogen deficiency and rhizobial symbiosis

PERMANOVA:

| **Source** | **DF** | **Sum of Squares** | **R2** | **F** | **p-value** |
| --- | --- | --- | --- | --- | --- |
| modality | 1 | 0.012912 | 0.04748 | 0.9969 | 0.4062 |
| residuals | 20 | 0.259034 | 0.95252 |  |  |
| total | 21 | 0.271946 | 1.00000 |  |  |

PERMDISP2:

| **Source** | **DF** | **Sum of Squares** | **Mean of Squares** | **F** | **p-value** |
| --- | --- | --- | --- | --- | --- |
| groups | 1 | 0.001313 | 0.0013129 | 0.6955 | 0.4166 |
| residual | 20 | 0.037757 | 0.0018878 |  |  |
