# Supplementary material for: Small impact of the host plant physiological state on the genome formula of a nanovirus
Source: PLoS Pathog. 2026 Sep 21;22(9):e1014641. doi: 10.1371/journal.ppat.1014641 (PMC13614657; doi:10.1371/journal.ppat.1014641)
Supplement: Supplementary file 2 [file ppat.1014641.s002.docx]

**Fig A. Shoot size of FBNSV-infected *V. faba* watered with deionized water (blue) or NaCl solution (red).** Plant shoot size was measured before sampling at 21 dpi. In boxplots, the central bar represents the median and the edges of the rectangle the first and third quartiles. The outer bars delineate 1.5 times the distance between the 25^th^ and 75^th^ percentile of the distribution. The dots represent the outliers. No statistically significant difference was found between the two conditions based on ANOVA test on non-transformed data (p-value=0.085; also see Table C in S1 Appendix).

**Fig B. Light intensity measured at the height of FBNSV-infected *V. faba*.** Light was maintained at “standard intensity” (green) until virus inoculation. After agro-inoculation, plantlets were placed under “higher light intensity” (blue) or “low light intensity” (red) in the same growth chamber. The light intensity (Photosynthetic Photon Flux Density; PPFD) at plant height was measured after inoculation (0 dpi), at mid-growth (11 dpi) and DNA sampling time (19 dpi) using a spectrophotometer. The plant height corresponds to the average height of the plants for each light intensity condition (0 dpi: 6 cm; 11dpi: 23.11 cm for high light intensity, 30.60 cm for low light intensity; 19 dpi: 32.71 cm for high light intensity and 39.57 cm for low light intensity). In boxplots, the central bar represents the median of the five measurement points (at the center and four sides of the shelf) and the edges of the rectangle the first and third quartiles. The outer bars delineate 1.5 times the distance between the 25^th^ and 75^th^ percentile of the distribution.

**Fig C. Shoot size (A), total fresh weight (B) and root fresh weight (C) of FBNSV-infected *V. faba* grown under nitrogen deficiency (blue) or rhizobial symbiosis (red).** Plant shoot size (A), shoot fresh weight (Fig 1F), total fresh weight (B) and root fresh weight (C) were determined before DNA extraction, 21 days after FBNSV inoculation. Total fresh weight was obtained by adding the shoot and root weights. In boxplots, the central bar represents the median and the edges of the rectangle the first and third quartiles. The outer bars delineate 1.5 times the distance between the 25^th^ and 75^th^ percentile of the distribution. The dots represent the outliers. Statistically significant differences between conditions were assessed on non-transformed data based on ANOVA (p≤0.05). While differences in total fresh (B) weight and shoot fresh weight (Fig 1F) could be observed, no statistically significant difference was found for shoot size (A; p-value=0.4507) and root fresh weight (C; p-value=0.1745) (also see Tables C and D in S1 Appendix).
