# Supplementary material for: Small impact of the host plant physiological state on the genome formula of a nanovirus
Source: PLoS Pathog. 2026 Sep 21;22(9):e1014641. doi: 10.1371/journal.ppat.1014641 (PMC13614657; doi:10.1371/journal.ppat.1014641)
Supplement: Supplementary file 3 [file ppat.1014641.s004.docx]

**Point-by-point response to the reviewers’ comments**

(reviewers’ comments are in small italics while our responses are in blue normal text)

We wish to thank the reviewer for the positive comments as well as for prompting us to speculate on the significance of our results. It is rare and very welcome.

Reviewer #1:

Minor comments:

*1- Abstract (lines 15–29): I suggest giving greater emphasis to the observation that changes in viral accumulation do not necessarily coincide with changes in genome formula. In particular, the finding that aphid infestation significantly reduces FBNSV accumulation while leaving the genome formula essentially unchanged is, in my opinion, one of the most informative outcomes of the study and deserves to be highlighted.*

We have revised the abstract to indicate that FBNSV accumulation is affected by aphid infestation (lines 27 to 29). As discussed by the reviewer in comment 7 and in our response to it, we agree with the reviewer that this stress probably reduces the amount of plant tissue available to the virus, and hence the number of infection foci, with no need to modulate viral gene expression and thus the genome formula.

*2- The Author Summary (lines 31–42) clearly explains the rationale of the study and summarizes the main findings. Even if the contrast between the marked genome formula remodeling observed following host switching (or during vector transmission) and the limited effect of host physiological changes is already presented later in the manuscript, I think it would be worth briefly recalling it in the final paragraph of the Author Summary, as it represents the main biological message of the study.*

We have supplemented the Author Summary to highlight the contrast between the drastic change of the genome formula occurring when switching hosts and the limited effect of host physiological changes (lines 43 to 44).

*3- Materials and Methods – Aphid infestation (lines 209–216): For reproducibility, it would be helpful to report the approximate aphid population present at the time of sampling.*

We added more details regarding aphid population at the time of sampling (lines 215 to 217). We unfortunately did not collect quantitative data on the precise size of aphid populations.

*4- Figure 2: The information required to interpret total FBNSV accumulation is clearly provided in the Materials and Methods. For the reader's convenience, I suggest to include this information also in the legend.*

We modified the legend of Figure 2 to include the information to interpret total FBNSV accumulation.

*5- Figures 1 and 2: The meaning of the asterisks indicating statistical significance is not explained in the legends. I suggest explicitly defining the significance threshold(s) corresponding to each symbol. If all asterisks simply denote statistical significance at the same threshold, a single asterisk would seem sufficient.*

We thank Reviewer #1 for pointing out the omission regarding the meaning of the asterisks indicating statistical significance in the legends of Figure 1 and Figure 2. We have added this information and explicitly defined the significance threshold. It so happens that all tests which were statistically significant had a p-value <0.001. We opted to use three stars to signal them since it is the standard practice for such p-values.

*6- Discussion (lines 329–339): The comparison with the study by Boezen et al. is interesting. The authors already acknowledge -that methodological and biological differences between the two studies make direct comparisons difficult. It would nevertheless be interesting if the authors could elaborate slightly on the biological differences between the two systems. Since the rationale of the present study is based on the analogy between genome formula plasticity and copy number variation as a mechanism to modulate viral gene expression, it would be valuable to discuss whether this analogy is expected to apply equally to multipartite RNA and ssDNA viruses. In particular, because genome segments of multipartite RNA viruses also serve directly as messenger RNAs, whereas FBNSV genome segments must first be transcribed by the host, the relationship between genome formula and viral gene expression may not necessarily be equivalent in the two systems. I recognize that this is a research article rather than an opinion piece, and therefore such considerations would necessarily remain speculative. Nevertheless, if the authors have a view on whether these biological differences could influence the relationship between genome formula and viral gene expression, I believe that a brief discussion would provide valuable context for interpreting the comparison.*

Indeed, in (+)ssRNA viruses genome segments serve directly as messenger RNAs. We did not want to insist on this aspect initially, because in our opinion it represents a weakness of these systems with respect to genome formula estimation. Indeed, as acknowledged by Boezen et al, because of this feature we do not know whether what is quantified is the relative accumulation of transcripts or the relative accumulation of genome segments. Most possibly it is a mixture of both in unknown proportions. It is thus very difficult to make a direct comparison of our results to results on such viruses. With hindsight, we should have acknowledged this in the first submission; we did not because we did not want to sound too critical to the work of our colleagues. We do this now (lines 377-384), and wish to thank the reviewer for prompting us to.

*7- Discussion (lines 352–359): In my opinion, the aphid infestation experiment is particularly informative because it is the only condition in which viral accumulation is significantly reduced while the genome formula remains essentially unchanged. The authors conclude that "no adjustments of FBNSV gene expression through copy number variation could alleviate or counteract the specific plant defense elicited by aphids" (lines 357–359). I find this interpretation somewhat problematic because no detectable adjustment in genome formula is observed in the first place. The statement therefore seems to imply the failure of a compensatory mechanism, whereas the data simply show that no genome formula remodeling occurred. Rather than suggesting that genome formula adjustment failed to compensate for the reduction in viral accumulation, the result may indicate that viral accumulation and genome formula are not necessarily tightly coupled. On the other hand, if genome formula acts as a mechanism to coordinate viral gene expression during infection, some relationship between genome formula and viral accumulation might be expected. The apparent lack of such a relationship may therefore reflect the scale at which viral accumulation was assessed, namely at the whole-host level rather than at local replication sites. An alternative possibility, consistent with the authors' suggestion that aphids may affect virus movement and the overall extent of infection, is that aphid infestation primarily reduces the extent of infected tissues, while the local viral load and replication dynamics at each replication site remain largely unchanged.* *In this scenario, the reduction in viral accumulation measured at the whole-plant level would simply reflect the sum of fewer infection sites, whereas the local conditions under which genome formula is established would remain essentially unchanged. Consequently, no genome formula remodeling would necessarily be expected. Discussing this alternative interpretation would broaden the biological significance of the results and help clarify whether changes in viral accumulation are mechanistically linked to genome formula remodeling.*

We have reworded our conclusion regarding the decrease in viral load with no impact on the genome formula observed during aphid infestation to hopefully remove the impression of a failure of a compensatory mechanism. We also thank Reviewer #1 for the interesting hypothesis regarding the decrease in total virus accumulation. We have added a few lines (lines 398 to 406 with references 53 and 54 lines 631 to 634) to discuss this interpretation.

*8- Discussion (lines 368–399): The authors argue that the hypothesis that the tested physiological conditions do not interfere with FBNSV infection "cannot be excluded but appears poorly satisfactory" (lines 368–369) and support this interpretation with several examples from the literature describing interactions between plant stress responses and virus infection. I find this reasoning convincing. However, the evidence that the applied stresses altered the environment experienced by FBNSV remains indirect, as it relies mainly on previous studies together with the whole-plant phenotypic characterization presented in Fig. 1 and the Supporting Information. The authors also mention that viral transcript accumulation was not analyzed. Given their previous work showing that the accumulation of FBNSV messenger RNAs correlates with the accumulation of the corresponding genome segments, suggesting that genome formula modulates viral gene expression (Bonnamy et al., 2024), I wonder what they would expect in the present system. Would the relative abundance of viral transcripts also remain unchanged, in agreement with the stable genome formula, or could host physiological perturbations affect viral transcript accumulation even when genome formula does not change? If the latter is considered possible, it would suggest that viral gene expression can respond to host physiology without genome formula remodeling. It would also argue against a coordinated regulatory mechanism linking viral transcript abundance to genome segment replication and, ultimately, genome formula. On the other hand, if transcript accumulation is also expected to remain unchanged, this would further support the idea that genome formula remodeling depends on biological differences associated with the host itself rather than on changes in its physiological state, helping to explain why genome formula remodeling is observed following host switching but not under the conditions examined in the present study. I appreciate that this is a research article rather than a Current Opinion, and therefore I do not expect the authors to discuss these questions in detail. Nevertheless, I think that briefly outlining these alternative scenarios would help place the present findings in a broader context and may stimulate future work aimed at understanding what ultimately determines genome formula plasticity in multipartite DNA viruses.*

We added text in the end of our hypothesis (i) (lines 446-452) we hope better acknowledging the two alternatives proposed by the reviewer: (1) mRNA formula does not change, or (2) it does change, and pointing to their implications, which were already mentioned in the Discussion.
