# Supplementary material for: Small impact of the host plant physiological state on the genome formula of a nanovirus
Source: PLoS Pathog. 2026 Sep 21;22(9):e1014641. doi: 10.1371/journal.ppat.1014641 (PMC13614657; doi:10.1371/journal.ppat.1014641)
Supplement: Peer review history

# Peer review: Decision Letter 0

**DOI:** 10.1371/journal.ppat.1014641.r001

**Authors:** Shou-Wei Ding, Rosa Lozano-Durán

28 Jul 2026

PPATHOGENS-D-26-01325

Small impact of the host plant physiological state on the genome formula of a nanovirus

PLOS Pathogens

Dear Dr. Michalakis,

Thank you for submitting your manuscript to PLOS Pathogens. After careful consideration, we feel that it has merit but does not fully meet PLOS Pathogens's publication criteria as it currently stands. Therefore, we invite you to submit a revised version of the manuscript that addresses the points raised during the review process.

Please submit your revised manuscript by Sep 26 2026 11:59PM. If you will need more time than this to complete your revisions, please reply to this message or contact the journal office at plospathogens@plos.org. When you're ready to submit your revision, log on to https://www.editorialmanager.com/ppathogens/ and select the 'Submissions Needing Revision' folder to locate your manuscript file.

Please include the following items when submitting your revised manuscript:

* A letter that responds to each point raised by the editor and reviewer(s). You should upload this letter as a separate file labeled 'Response to Reviewers'. This file does not need to include responses to any formatting updates and technical items listed in the 'Journal Requirements' section below.

* A marked-up copy of your manuscript that highlights changes made to the original version. You should upload this as a separate file labeled 'Revised Manuscript with Track Changes'.

* An unmarked version of your revised paper without tracked changes. You should upload this as a separate file labeled 'Manuscript'.

If you would like to make changes to your financial disclosure, competing interests statement, or data availability statement, please make these updates within the submission form at the time of resubmission. Guidelines for resubmitting your figure files are available below the reviewer comments at the end of this letter.

As the corresponding author, your ORCID iD is verified in the submission system and will appear in the published article. PLOS supports the use of ORCID, and we encourage all coauthors to register for an ORCID iD and use it as well. Please encourage your coauthors to verify their ORCID iD within the submission system before final acceptance, as unverified ORCID iDs will not appear in the published article. Only the individual author can complete the verification step; PLOS staff cannot verify ORCID iDs on behalf of authors.

We look forward to receiving your revised manuscript.

Kind regards,

Rosa Lozano-Durán

Academic Editor

PLOS Pathogens

Shou-Wei Ding

Section Editor

PLOS Pathogens

Sumita Bhaduri-McIntosh

Editor-in-Chief

PLOS Pathogens

orcid.org/0000-0003-2946-9497

Michael Malim

Editor-in-Chief

PLOS Pathogens

orcid.org/0000-0002-7699-2064

**Additional Editor Comments (if provided):**

**Journal Requirements:**

If the reviewer comments include a recommendation to cite specific previously published works, please review and evaluate these publications to determine whether they are relevant and should be cited. There is no requirement to cite these works unless the editor has indicated otherwise.

1) We ask that a manuscript source file is provided at Revision. Please upload your manuscript file as a .doc, .docx, .rtf or .tex. If you are providing a .tex file, please upload it under the item type u2018LaTeX Source Fileu2019 and leave your .pdf version as the item type u2018Manuscriptu2019.

2) We do not publish any copyright or trademark symbols that usually accompany proprietary names, eg ©,  ®, or TM  (e.g. next to drug or reagent names). Therefore please remove all instances of trademark/copyright symbols throughout the text, including:

- ® on page: 23

- TM on pages: 10, and 18.

3) Please upload all main figures as separate Figure files in .tif or .eps format. For more information about how to convert and format your figure files please see our guidelines:

https://journals.plos.org/plospathogens/s/figures

4) When completing the data availability statement of the submission form, you indicated that you will make your data available on acceptance. We strongly recommend all authors decide on a data sharing plan before acceptance, as the process can be lengthy and hold up publication timelines. Please note that, though access restrictions are acceptable now, your entire data will need to be made freely accessible if your manuscript is accepted for publication. This policy applies to all data except where public deposition would breach compliance with the protocol approved by your research ethics board. If you are unable to adhere to our open data policy, please kindly revise your statement to explain your reasoning and we will seek the editor's input on an exemption. Please be assured that, once you have provided your new statement, the assessment of your exemption will not hold up the peer review process.

5) Please amend your detailed Financial Disclosure statement. This is published with the article. It must therefore be completed in full sentences and contain the exact wording you wish to be published.

i) State what role the funders took in the study. If the funders had no role in your study, please state: "The funders had no role in study design, data collection and analysis, decision to publish, or preparation of the manuscript."

ii) If any authors received a salary from any of your funders, please state which authors and which funders..

If you did not receive any funding for this study, please simply state: u201cThe authors received no specific funding for this work.u201d

**Reviewers' Comments:**

Reviewer's Responses to Questions

**Part I - Summary**

Please use this section to discuss strengths/weaknesses of study, novelty/significance, general execution and scholarship.

Reviewer #1: This manuscript investigates whether environmentally induced changes in host physiology influence the genome formula of the multipartite virus FBNSV. Building on previous studies showing that FBNSV genome formula undergoes substantial remodeling following host switching, and on the hypothesis that genome formula plasticity represents a mechanism analogous to copy number variation in cellular organisms for modulating gene expression in response to environmental changes, the authors investigate whether genome formula also undergoes remodeling in response to physiological perturbations within the same host species. Overall, the study is well designed, clearly presented, and supports the authors' conclusion that, unlike host switching, the physiological perturbations examined have little or no impact on FBNSV genome formula, even when viral accumulation is affected. I believe this is a solid and interesting study. Given the very limited number of studies investigating genome formula remodeling under conditions other than host switching, this work represents a valuable contribution. The comparison with the previous study on multipartite RNA viruses, already discussed by the authors, further highlights the relevance of these findings. The revisions I suggest are minor and are mainly intended to improve the interpretation and presentation of the results.

The statistical analyses appear appropriate to me. However, I do not consider myself sufficiently qualified to assess them in detail and therefore defer to reviewers with greater expertise in statistics.

**********

**Part II – Major Issues: Key Experiments Required for Acceptance**

Please use this section to detail the key new experiments or modifications of existing experiments that should be absolutely required to validate study conclusions.

Generally, there should be no more than 3 such required experiments or major modifications for a "Major Revision" recommendation. If more than 3 experiments are necessary to validate the study conclusions, then you are encouraged to recommend "Reject".

Reviewer #1: (No Response)

**********

**Part III – Minor Issues: Editorial and Data Presentation Modifications**

Please use this section for editorial suggestions as well as relatively minor modifications of existing data that would enhance clarity.

Reviewer #1: Abstract (lines 15–29): I suggest giving greater emphasis to the observation that changes in viral accumulation do not necessarily coincide with changes in genome formula. In particular, the finding that aphid infestation significantly reduces FBNSV accumulation while leaving the genome formula essentially unchanged is, in my opinion, one of the most informative outcomes of the study and deserves to be highlighted.

The Author Summary (lines 31–42) clearly explains the rationale of the study and summarizes the main findings. Even if the contrast between the marked genome formula remodeling observed following host switching (or during vector transmission) and the limited effect of host physiological changes is already presented later in the manuscript, I think it would be worth briefly recalling it in the final paragraph of the Author Summary, as it represents the main biological message of the study.

Materials and Methods – Aphid infestation (lines 209–216): For reproducibility, it would be helpful to report the approximate aphid population present at the time of sampling.

Figure 2: The information required to interpret total FBNSV accumulation is clearly provided in the Materials and Methods. For the reader's convenience, I suggest to include this information also in the legend.

Figures 1 and 2: The meaning of the asterisks indicating statistical significance is not explained in the legends. I suggest explicitly defining the significance threshold(s) corresponding to each symbol. If all asterisks simply denote statistical significance at the same threshold, a single asterisk would seem sufficient.

Discussion (lines 329–339): The comparison with the study by Boezen et al. is interesting. The authors already acknowledge that methodological and biological differences between the two studies make direct comparisons difficult. It would nevertheless be interesting if the authors could elaborate slightly on the biological differences between the two systems. Since the rationale of the present study is based on the analogy between genome formula plasticity and copy number variation as a mechanism to modulate viral gene expression, it would be valuable to discuss whether this analogy is expected to apply equally to multipartite RNA and ssDNA viruses. In particular, because genome segments of multipartite RNA viruses also serve directly as messenger RNAs, whereas FBNSV genome segments must first be transcribed by the host, the relationship between genome formula and viral gene expression may not necessarily be equivalent in the two systems. I recognize that this is a research article rather than an opinion piece, and therefore such considerations would necessarily remain speculative. Nevertheless, if the authors have a view on whether these biological differences could influence the relationship between genome formula and viral gene expression, I believe that a brief discussion would provide valuable context for interpreting the comparison.

Discussion (lines 352–359): In my opinion, the aphid infestation experiment is particularly informative because it is the only condition in which viral accumulation is significantly reduced while the genome formula remains essentially unchanged. The authors conclude that "no adjustments of FBNSV gene expression through copy number variation could alleviate or counteract the specific plant defense elicited by aphids" (lines 357–359). I find this interpretation somewhat problematic because no detectable adjustment in genome formula is observed in the first place. The statement therefore seems to imply the failure of a compensatory mechanism, whereas the data simply show that no genome formula remodeling occurred. Rather than suggesting that genome formula adjustment failed to compensate for the reduction in viral accumulation, the result may indicate that viral accumulation and genome formula are not necessarily tightly coupled. On the other hand, if genome formula acts as a mechanism to coordinate viral gene expression during infection, some relationship between genome formula and viral accumulation might be expected. The apparent lack of such a relationship may therefore reflect the scale at which viral accumulation was assessed, namely at the whole-host level rather than at local replication sites. An alternative possibility, consistent with the authors' suggestion that aphids may affect virus movement and the overall extent of infection, is that aphid infestation primarily reduces the extent of infected tissues, while the local viral load and replication dynamics at each replication site remain largely unchanged. In this scenario, the reduction in viral accumulation measured at the whole-plant level would simply reflect the sum of fewer infection sites, whereas the local conditions under which genome formula is established would remain essentially unchanged. Consequently, no genome formula remodeling would necessarily be expected. Discussing this alternative interpretation would broaden the biological significance of the results and help clarify whether changes in viral accumulation are mechanistically linked to genome formula remodeling.

Discussion (lines 368–399): The authors argue that the hypothesis that the tested physiological conditions do not interfere with FBNSV infection "cannot be excluded but appears poorly satisfactory" (lines 368–369) and support this interpretation with several examples from the literature describing interactions between plant stress responses and virus infection. I find this reasoning convincing. However, the evidence that the applied stresses altered the environment experienced by FBNSV remains indirect, as it relies mainly on previous studies together with the whole-plant phenotypic characterization presented in Fig. 1 and the Supporting Information. The authors also mention that viral transcript accumulation was not analyzed. Given their previous work showing that the accumulation of FBNSV messenger RNAs correlates with the accumulation of the corresponding genome segments, suggesting that genome formula modulates viral gene expression (Bonnamy et al., 2024), I wonder what they would expect in the present system. Would the relative abundance of viral transcripts also remain unchanged, in agreement with the stable genome formula, or could host physiological perturbations affect viral transcript accumulation even when genome formula does not change? If the latter is considered possible, it would suggest that viral gene expression can respond to host physiology without genome formula remodeling. It would also argue against a coordinated regulatory mechanism linking viral transcript abundance to genome segment replication and, ultimately, genome formula. On the other hand, if transcript accumulation is also expected to remain unchanged, this would further support the idea that genome formula remodeling depends on biological differences associated with the host itself rather than on changes in its physiological state, helping to explain why genome formula remodeling is observed following host switching but not under the conditions examined in the present study. I appreciate that this is a research article rather than a Current Opinion, and therefore I do not expect the authors to discuss these questions in detail. Nevertheless, I think that briefly outlining these alternative scenarios would help place the present findings in a broader context and may stimulate future work aimed at understanding what ultimately determines genome formula plasticity in multipartite DNA viruses.

**********

PLOS authors have the option to publish the peer review history of their article (what does this mean?). If published, this will include your full peer review and any attached files.

If you choose “no”, your identity will remain anonymous but your review may still be made public.

**Do you want your identity to be public for this peer review?**  For information about this choice, including consent withdrawal, please see our Privacy Policy.

Reviewer #1: No

[NOTE: If reviewer comments were submitted as an attachment file, they will be attached to this email and accessible via the submission site. Please log into your account, locate the manuscript record, and check for the action link "View Attachments". If this link does not appear, there are no attachment files.]

**Figure resubmission:**

**Reproducibility:**

To enhance the reproducibility of your results, we recommend that authors of applicable studies deposit laboratory protocols in protocols.io, where a protocol can be assigned its own identifier (DOI) such that it can be cited independently in the future. Additionally, PLOS ONE offers an option to publish peer-reviewed clinical study protocols. Read more information on sharing protocols at https://plos.org/protocols?utm_medium=editorial-email&utm_source=authorletters&utm_campaign=protocols

# Author comment: Author response to Decision Letter 1

**DOI:** 10.1371/journal.ppat.1014641.r002

3 Sep 2026

Attachment Submitted filename: response to reviewers final.docx

# Editor report: Decision Letter 1

**DOI:** 10.1371/journal.ppat.1014641.r003

**Authors:** Shou-Wei Ding, Rosa Lozano-Durán

10 Sep 2026

Dear Dr. Michalakis,

We are pleased to inform that your manuscript, "Small impact of the host plant physiological state on the genome formula of a nanovirus", has been editorially accepted for publication at PLOS Pathogens.

Before your manuscript can be formally accepted and sent to production, you will need to complete our formatting changes, which you will receive by email within a week. Please note that your manuscript will not be scheduled for publication until you have made the required changes.

IMPORTANT NOTES

(1) Copyediting and Proofreading: The corresponding author will receive a typeset proof for review, to ensure errors have not been introduced during production. Please review the PDF proof of your manuscript carefully, as this is the last chance to correct any errors. Please note that major changes, or those which affect the scientific understanding of the work, will likely cause delays to the publication date of your manuscript.

(2) Appropriate Figure Files: Please remove all name and figure # text from your figure files. Please also take this time to check that your figures are of high resolution, which will improve the readbility of your figures and help expedite your manuscript's publication. Please note that figures must have been originally created at 300dpi or higher. Do not manually increase the resolution of your files. For instructions on how to properly obtain high quality images, please review our Figure Guidelines, with examples at: http://journals.plos.org/plospathogens/s/figures.

We encourage you to use PACE (http://pace.apexcovantage.com/), a figure-checking tool which helps authors ensure all figures meet PLOS requirements so that figures can publish in the highest quality. PACE is a digital diagnostic and conversion tool for figure files. It will provide information about any failed check(s) and, if able, will automatically convert the figure into an acceptable file that passes quality checks.

To use PACE, you must first register as a user. Then, login and navigate to the UPLOAD tab, where you will find detailed instructions on how to use the tool.

(3) Striking Image: Please upload a striking still image to accompany your article if one is available (you can include a new image or an existing one from within your manuscript). Should your paper be accepted, this image will be considered for our monthly issue image and may also appear on our website to feature your article. Please upload this as a separate file, selecting "striking image" as the file type upon upload. Please also include a separate "Other" file with a caption, including credits and any potential copyright information. Please do not include the caption in the main article file. If your image is from someone other than yourself, please ensure that the artist has read and agreed to the terms and conditions of the Creative Commons Attribution License at http://journals.plos.org/plospathogens/s/content-license. Please note that PLOS cannot publish copyrighted images.

(4) Press Release or Related Media: If you or your institution will be preparing press materials for this manuscript, please inform our press team in advance at plospathogens@plos.org as soon as possible. We ask that you contact us within one week to plan ahead of our fast Production schedule. If you need to know your paper's publication date for related media purposes, you must coordinate with our press team, and your manuscript will remain under a strict press embargo until the publication date and time.

(5) Update your Profile Information: Now that your manuscript has been provisionally accepted, please log into Editorial Manager and update your profile, if needed. Go to http://www.editorialmanager.com/ppathogens, log in, and click on the "Update My Information" link at the top of the page. Please update your user information to ensure an efficient production and billing process.

(6) LaTeX users only: Our staff will ask you to upload a TEX file in addition to the PDF before the paper can be sent to typesetting, so please carefully review our Latex Guidelines http://journals.plos.org/plospathogens/s/latex in the meantime.

Thank you again for supporting Open Access publishing; we are looking forward to publishing your work in PLOS Pathogens.

Best regards,

Rosa Lozano-Durán

Academic Editor

PLOS Pathogens

Shou-Wei Ding

Section Editor

PLOS Pathogens

Dominique Soldati-Favre

Editor-in-Chief

PLOS Pathogens

***********************************************************

# Editor report: Acceptance letter

**DOI:** 10.1371/journal.ppat.1014641.r004

**Authors:** Shou-Wei Ding, Rosa Lozano-Durán

Dear Dr. Michalakis,

We are delighted to inform you that your manuscript, "Small impact of the host plant physiological state on the genome formula of a nanovirus," has been formally accepted for publication in PLOS Pathogens.

We have now passed your article onto the PLOS Production Department who will complete the rest of the pre-publication process. All authors will receive a confirmation email upon publication.

Thank you again for supporting open-access publishing; we are looking forward to publishing your work in PLOS Pathogens.

Best regards,

Dominique Soldati-Favre

Editor-in-Chief

PLOS Pathogens
